# Revision TKA with a condylar constrained prosthesis using metaphyseal and surface cementation: a minimum 6-year follow-up analysis

**DOI:** 10.1186/s12891-015-0485-6

**Published:** 2015-02-25

**Authors:** Pablo Sanz-Ruiz, Manuel Villanueva-Martínez, Jose Antonio Matas-Diez, Javier Vaquero-Martín

**Affiliations:** From the Department of Traumatology and Orthopaedic Surgery, General University Hospital Gregorio Marañón, Doctor Esquerdo Street, number 46, ZIP code 28007 Madrid, Spain

**Keywords:** Revision TKA, Constrained condylar, Fixation, Press-fit stem

## Abstract

**Background:**

The increasing number of revision knee arthroplasty result in the more frequently use of a constraint implant but results from previous reports are difficult to interpret. The purpose of this study was to compare the long-term outcomes of superficial cemented versus metaphyseal cemented in revision total knee arthroplasty with a condylar constrained arthroplasty.

**Methods:**

The study was a retrospective analysis of clinical and radiographic outcomes in a series of revision total knee arthroplasties performed with a constrained condylar knee prosthesis and press-fit modular stems. We hypothesized that the clinical and radiographic outcome of surface cementation would be inferior to that of metaphyseal cementation. Fifty-two consecutive revision cases were followed for a median of 8.2 years (range, 6 to 10 years).

**Results:**

Substantial improvements in range of motion and Knee Society score were achieved in all patients, although these were not significant between groups. Significantly more radiolucent lines were visible on the tibial component with surface cementation than with metaphyseal cementation, although the clinical differences were not relevant.

**Conclusions:**

Radiologic outcome was better in revision total knee arthroplasty using metaphyseal cemented revision and components with press-fit cementless stems than in the surface cementation–based approach; however, the difference was not clinically relevant.

## Background

The increasing number of patients undergoing primary total knee arthroplasty (TKA) has been accompanied by an increase in the number of revision knee arthroplasty procedures, with an estimated cost of €50,000-70,000 per procedure [1,2]. The goal of revision knee arthroplasty is to restore the anatomy and function of the joint. However, bone loss and soft tissue instability are substantial obstacles to obtaining adequate joint line restoration [3] and ligament balancing, which are crucial when deciding on a suitable choice of implant. The constrained condylar knee (CCK) prosthesis was developed to resist coronal plane stresses arising from soft-tissue instability.

Results from previous reports of patients treated with modern CCK prostheses are difficult to compare, because different types of prostheses were used and follow-up periods were fairly short. In this report, we aim to provide a more robust clinical and radiographic evaluation of the CCK arthroplasty procedures performed in our institution between 2004 and 2008 using the same type of prosthesis for patients with instability or severe bone loss. We evaluated the functional outcome of the knees, radiographic results, and potential complications.

## Methods

All patients gave their informed consent before inclusion in the study. Ours was a retrospective study performed in accordance with the principles of the 1964 Declaration of Helsinki as revised in 2013 and was approved by the research ethics committee of the Gregorio Marañon General Hospital.

**Figure 1:**
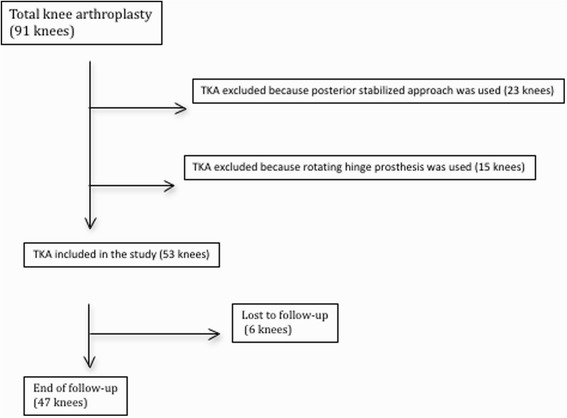
**Flowchart showing inclusion criteria of all total knee revision.**

Between January 2004 and December 2007, 91 consecutive revision total knee arthroplasties were performed in 88 patients by 2 senior authors (MVM and JVM) in a single institution using the same system. Twenty-three procedures were excluded because a posterior stabilized liner was used with a CCK prosthesis, and 15 were excluded because the revision was performed using a rotating hinge knee prosthesis (NexGen® RH Knee, Zimmer, Warsaw, Indiana, USA) (Figure 1). The implant chosen in all cases was the Legacy Constrained Condylar Knee prosthesis (LCCK; Zimmer, Warsaw, Indiana, USA). In the remaining 53 procedures, the indication for using this prosthesis was an absent posterior cruciate ligament and a deficient medial or lateral collateral ligament but a competent extensor mechanism. According to the Anderson Orthopaedic Research Institute [4] bone defect classification, there were 19 type 1 femoral defects, 32 type 2 defects, and 2 type 3 defects. As for tibial defects, 29 were type 1, 20 were type 2, and 4 were type 3. Both aseptic and septic cases were included.

Data from the 53 procedures were entered into a computerized registry. Data on all the knees were reviewed by an orthopedic surgeon who was not involved in the original procedure. Clinical and radiologic follow-up evaluations were planned at postoperative intervals of 1 month, 3 months, 6 months, and 1 year, and yearly thereafter. Preoperative and postoperative review data were collected according to the scoring systems of the Knee Society [5].

Normotensive epidural anesthesia was used in all the procedures. All the knees were approached through a previous anterior midline incision scar. A quadriceps snip [6] was required in 13 knees, and a tibial tubercle osteotomy in 3. All the osteotomies and 5 snips were performed in septic cases during the second stage.

**Figure 2:**
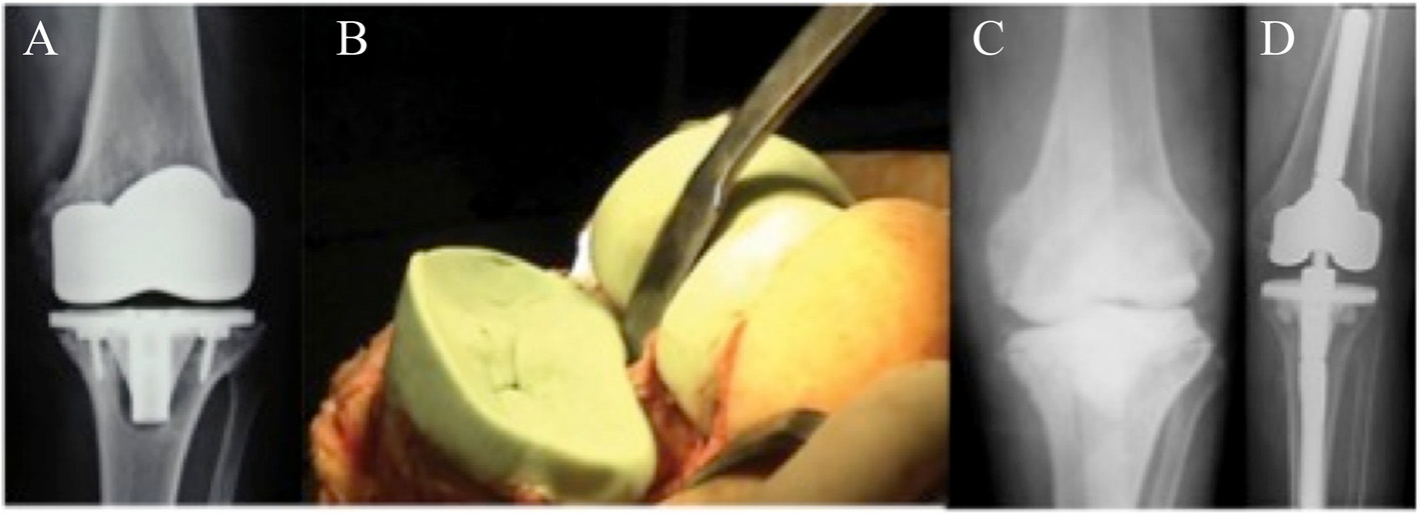
**74 y, male: 4 years after TKA with septic loosening and medial tibial collapse A) preoperative x-ray B) Two stage revision with hand made antibiotic-loaded articulating cement spacer. C)** Postoperative x-ray a.p. view **D)** Second stage revision after an interval of 8 weeks with condylar constraint prosthesis.

Surgical technique: Failed components were removed while preserving as much bone stock as possible, and extensive debridement was performed. The components were cleaned with saline solution. In all cases, at least 5 intraoperative samples were obtained for microbiological culture. Both medullary canals were reamed to the point of mild resistance, but not until the so-called cortical chatter was detected. The osseous surfaces were then meticulously prepared with a saw or a high-speed burr to increase surface contact area. The tibial surface was reconstructed first. After tibial reconstruction, flexion space was balanced to ensure correct femoral size. The size of the femoral components was planned using the contralateral knee X-rays and the charts of the primary TKA. Correct positioning of the prosthetic joint line was determined using preoperative radiographs, by measuring the femoral width multiplied by a constant (0.27 for all patients), which gives the distance from the proximal limit of the tibial tubercle to the joint line, as previously described by Servien et al. [7]. Two groups were defined according to the cementing technique used. Palacos R + G (Heraeus Medical GmbH, Wehrheim, Germany) manually mixed with 1 g of extra vancomycin was used in both groups. In one group, the cement was pressed into the bone surface using the fingers (surface cementation), and the modular stem was fixed by press fitting; in the other group, cement was used both on the bone surface and around the metaphyseal bone near the modular stem (metaphyseal cementation), and the modular stem was fixed by press fitting, as in the surface cementation group. The patella was resurfaced at the time of revision surgery in 10 knees (23.4%). All infected cases were treated with a 2-stage exchange arthroplasty using a hand made articulate cement spacer with antibiotic [8,9] (Figure 2). Routine prophylaxis with cefazolin was applied for 24 hours in aseptic cases. In infected cases, the hospital protocol (teicoplanin and meropenem) was administered until negative cultures were obtained.

Tibial augments were needed in 20 cases (42) (14 aseptic [45%] and 6 infected [60%]), and femoral augments were needed in 32 cases (68%) (24 aseptic [77%] and 8 infected [80%]). Tantalum cones were necessary in 4 cases (all septic). The length and diameter of the femoral and tibial stems are shown in Table 1. The cementing technique was superficial in 29 cases (62%) and metaphyseal in 18 cases (38%). The mean tourniquet time was 115 minutes (68 to 200).

**Table 1:** **Implant data**

	**Septic group**	**Aseptic group**	**P value**
	**n = 10**	**n = 37**	
Stem			
Femoral	13.5 (11–18)	13 (11–16)	0.26
Tibial	11.8 (11–15)	12.5 (11–15)	0.18
Metal augment			
Femoral	8	24	0.64
Tibial	6	14	0.233
Tantalum cones	4	0	
Offset			
Femoral	3	5	0.27
Tibial	8	19	0.122

**Figure 3:**
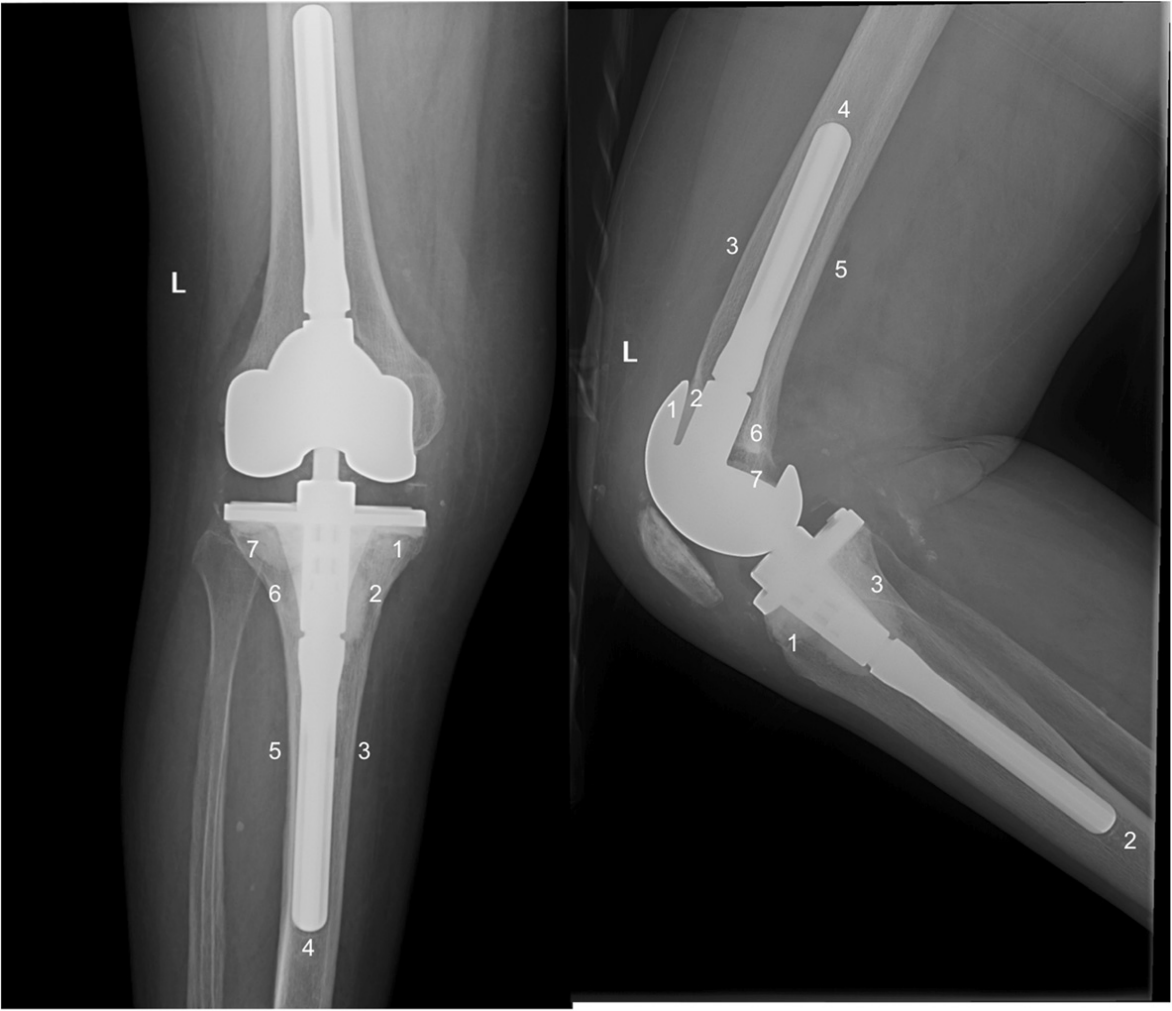
**Radiographic zones for the evaluation of the radiolucent lines.**

Standing anteroposterior radiographs including the femoral head and ankle, as well as supine, lateral, and skyline radiographs of the patella, were obtained under fluoroscopic guidance. An independent observer, who was not involved in the procedure, assessed the radiographs for the alignment of the limb, the position of the components, and the presence and location of all radiolucent lines at the cement–bone interface (Figure 3), according to the recommendations of the Knee Society [10].

The functional and clinical results were evaluated according to the Knee Society Score and compared with the radiographic and demographic variables mentioned above.

### Statistical analysis

Statistical analysis was performed using SPSS 20.0 for Windows (IBM Corp., Armonk, New York, USA). All data were checked for normality using the Shapiro-Wilk W test. The *t* test was used to compare scores for the normal distribution between the preoperative and postoperative data. The degree of satisfaction between the 2 groups was compared using the chi-square test. Statistical significance was set at p <0.05. Kaplan-Meier curves were used to analyze the survival of the prostheses.

## Results

Six out of the 53 revisions performed with the LCCK system were lost to follow-up. The final sample comprised 47 TKAs (23 right knees and 24 left knees in 47 patients). Mean age was 73 years (59 to 85 years), and 40 patients were women. The indication for revision was aseptic loosening in 31, deep infection in 10, ligament instability in 4, fracture in 1, and implant failure in 1 (Table 2). Average preoperative and postoperative flexion, extension, ranges of motion, and knee and functional score according to the Knee Society system are shown in Table 3. The preoperative range of movement and Knee Society scores were worse in septic cases

**Table 2:** **Demographic and clinical data**

Gender	
M/F	7/40
Age (y)	73.17 (59–85)
Side	
R/L	25/22
Primary diagnosis, No. of knees (%)	
Osteoarthritis	42 (89.4)
Rheumatoid arthritis	5 (10.6)
Reason for revision, number of knees (%)	
Aseptic loosening	31 (65.9)
Infection	10 (21.3)
Instability	4 (8.4)
Periprosthetic fracture	1 (2.1)
Implant failure	1 (2.1)
Prosthesis before revision (no [%] of knees)	
Profix	12 (25.5)
Nex-Gen	10 (21.3)
Tricon	8 (17)
CKS	5 (10.6)
Insall-Burstein	5 (10.6)
Alienor	2 (4.2)
LCCK	2 (4.2)
Excel	1 (2.1)
Genesis I	1 (2.1)
Milles Galante	1 (2.1)
Mean duration between primary and revision TKAs (months) (range)	97 (0–240)
Duration of follow-up (months) (range)	98.4 (72–120)

**Table 3:** **Clinical results at the end of follow-up**

**Parameter**	**Mean values**	**P value**
FCA (range)		
Preoperative	4.4 (−15 to 30)	<0.001
Postoperative	2.44 (0 to 15)	
MFA (range)		
Preoperative	90.4 (60 to 120)	<0.001
Postoperative	105.5 (70 to 130)	
Range of movement (range)		
Preoperative	86 (30 to 120)	<0.001
Postoperative	108 (70 to 130)	
Knee Society knee score (points) (range)		
Preoperative	39.4 (0 to 67)	<0.001
Postoperative	78.7 (45 to 100	
Knee Society function score (points) (range)		
Preoperative	32.1 (0 to 73)	<0.001
Postoperative	56 (5 to 100)	

FCA, flexion contracture angle; MFA, maximum flexion angle.

The Knee Society score increased in all patients after revision surgery. At 1 year, the clinical score had increased by a mean of 42.9 (14 to 74); however, at the end of follow-up this value had decreased by a mean of 3.6 points. No statistical differences were found between the time points. Clinical outcome was rated as excellent in 21 revisions (44.7%), good or moderate in 18 (38.3%), and poor in 8 cases (17%). The functional score increased by a mean of 29.3 (5 to 85) at 1 year after surgery; however, at the end of follow-up it had decreased by a mean of 5.4 (−30 to +40) (Table 3). No statistical differences were detected between the scores at 1 year or at the end of follow-up, when the score for function was excellent in 18 revisions (38.3%), good or moderate in 19 (40.4%), and poor in 10 cases (21.3%). No differences were observed in the Knee Society scores between aseptic and septic cases (p = 0.4) or between the different cementing techniques used.

**Figure 4:**
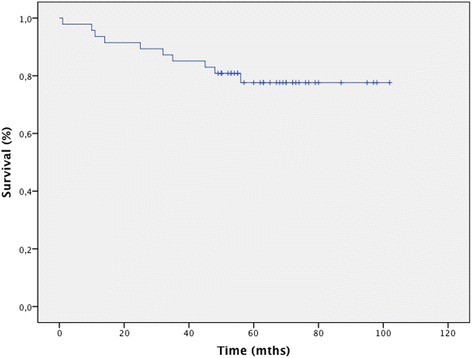
**Survival curve for the 49 revision knee arthroplasty procedures at final follow-up.**

Radiographic outcome at the end of follow-up is shown in Tables 4 and 5. Radiolucent lines (defined as more than 1 mm) [10] at the bone cement interface were identified in 23 procedures (48.9%). At the last follow-up visit, only 2 (6%) of these lines had progressed. Radiolucent lines were observed on the lateral femoral radiographs in 15 knees and on the tibial radiograph in 16 knees (13 knees in the lateral views and 16 knees in the anteroposterior views). No differences in radiolucency were observed between septic and aseptic cases (p = 0.8). A significantly higher frequency of radiolucent lines was observed for surface cementation on the tibial component than for metaphyseal cementation (p = 0.04). No significant differences were observed in the femoral components. The projected rate of survival at 9 years was 80% (95% confidence interval [CI], 76% to 95%) using revision surgery as an endpoint of follow-up (Figure 4).

**Table 4:** **Radiographic results at the end of follow-up**

**Parameter**	**Before revision**	**After revision**	**P value**
Tibiofemoral angle	6 (0 to 20)	2.97 (0 to 6)	0.003
Femoral angle			
Coronal	94.3 (86 to 104)	97.6 (87 to 100)	<0.001
Sagittal	5.2 (−3 to 12)	4.17 (0 to 10)	0.067
Tibial angle			
Coronal	85.4 (80 to 91)	87.4 (85 to 93)	<0.001
Sagittal	88.2 (81 to 94)	84.2 (81 to 87)	<0.001
Joint line (cm)	1.4 (0.9 to 2.1)	1.6 (0.8 to 2.3)	0.029

**Table 5:** **Radiolucency around the implants**

	**Zone 1**	**Zone 2**	**Zone 3**	**Zone 4**	**Zone 5**	**Zone 6**	**Zone 7**	**Total**
Lat femur	5 (11%)	4 (8.5%)	4 (8.5%)	4 (8.5%)	8 (17%)	7 (15%)	7 (14.9%)	23 (49%)
AP tibia	10 (21%)	10 (21%)	11 (23%)	12 (25%)	8 (17%)	9 (19%)	5 (11%)	16 (34%)
Lat tibia	7 (15%)	8 (17%)	11 (23%)	-	-	-	-	13 (28%)

There were 11 complications. Intraoperative complications included 3 partial avulsions and 1 total avulsion of the patellar tendon that were treated successfully by anchor fixation and 1 condylar fracture that was fixed with screws. Postoperative complications included 2 infections, 2 periprosthetic fractures treated with plate synthesis, 1 pulmonary embolism, and 1 postoperative hematoma requiring surgical drainage (Table 6). Three revisions were necessary. Two were because of infection (1 reinfection and 1 new infection): one was treated with 2-stage revision and the other with arthrodesis with a cemented nail due to massive bone loss. The third revision involved a case of severe mediolateral instability that was treated with revision of a rotating hinge knee prosthesis (NexGen®, Zimmer, Warsaw, Indiana, USA) at 15 months after surgery. The follow-up was 8.2 years (6 to 10).

**Table 6:** **Complications**

**Complication**	**n**	**%**
Rupture of extensor apparatus (intraoperative)	4	8.5
Total	1	
Partial	3	
Condylar fracture (intraoperative)	1	2.1
Infection	2	4.2
Periprosthetic fracture	2	4.2
Pulmonary embolism	1	2.1
Postoperative hematoma	1	2.1

## Discussion

Favorable clinical outcomes with revision condylar constrained TKA have been reported by several authors and include improved quality of life, pain relief, increased walking distance, reduced deformity, and enhanced function [11-16]. However, comparison of clinical outcome and complications is difficult, as is comparison of condylar-constrained TKA studies in the literature because of the different types of prostheses and the relatively short follow-up periods.

Hossain et al. [17] reported the clinical results of 349 revision TKAs after a mean follow up of 57.7 months. The authors compared 3 different implant designs and found that functional outcome and range of motion improved significantly, irrespective of the implant used. Kim et al. [18] reported the clinical results of 114 LCCK-based revision TKAs after a follow-up of 7.2 years. Of the 114 knees studied, clinical improvement was achieved in 91%, and radiographically stable implant fixation was observed in 96%. The mean range of motion was 95° before surgery and 106° at the end of follow-up; however, both posterior stabilized and CCK liners were used. Harwin [19] reported excellent results with the 18 Kinemax Plus Superstabilizer prosthesis after a follow-up of 11.3 years. Septic revision, supracondylar fracture, and neuropathic arthropathy were excluded in this series.

In our study, the mean Knee Society clinical score of the 47 patients at the end of follow-up (mean, 6.5 years) was 78.7 (45 to 100); 39 patients (83%) had an excellent or good result. Our findings are comparable to those reported by Lee et al. [20] in the longest follow-up of a single prosthesis used for revision TKA with only a CCK liner. The series contained roughly similar numbers of septic and aseptic revisions.

Several clinical studies have suggested that radiolucent lines are more frequent after revision TKA than after primary TKA [21,22]. One early clinical study of revision TKA using the Total Condylar-III prosthesis found radiolucent lines in 22 of 36 patients (61%) [13]. In another series of 14 TKAs, radiolucent lines were identified around the tibial component in 10 knees and around the femoral component in 4 knees [11]. In 2 recent studies, radiolucent lines were observed in 36% (13 out of 36 knees) to 72% (28 out of 39 knees) after revision TKA using different system designs [23,24]. Kim et al. [18] identified radiolucent lines around the tibial component in 31% (35 out of 114 knees) and in 18% (21 knees) on the femoral side, although most implants were posterior stabilized. In the present study, radiolucent lines were identified in 49% (23), 34% (16) around the tibial component, especially in zone 3 in the lateral tibial view and zone 4 in the anteroposterior tibial view, and in 17% (8) around the femoral component, especially in zone 5 in the lateral femoral view.

The ideal method of fixation in revision TKR remains controversial. The use of cement for at least a portion of the femoral and tibial components is well accepted in most procedures; however, the issue of whether to cement the stemmed portion of the implant is not clear [25]. Cemented fixation has the advantage that antibiotics can be added to the cement [26], although its disadvantages include difficulty in removing cement at re-revision (if necessary) and the potential for stress shielding [27]. Murray et al. [28] showed sclerotic lines around long cemented stems and similar trabecular patterns around the stem in cemented and non-cemented stems at 5 years of follow-up and concluded that there was no stress shielding. Completo et al. [27], on the other hand, recently demonstrated stress shielding in cemented stems *in vitro*. Marx et al. [29] recommended surface cementation to avoid stress shielding. To our knowledge, there are no reports directly comparing the cementation technique with hybrid fixation in revision TKA.

In our series, we observed a higher proportion of radiolucent lines at the bone-cement interface with surface cementation than with metaphyseal cementation. These results are consistent with those published by Conlisk et al. [30] in an in vitro study that showed better initial stability when more cement was used. Wood et al. [31] reported 87% survival with metaphyseal cementation and press-fit stem at 12 years of follow-up in 127 patients. Our findings (80%) are consistent with those of other series. Lee et al. [32] reported a survival rate of 83% at 8 years in 79 revisions, and Peters et al. [25] reported a rate of 80% at 80 months in 43 revisions. This study showed a survival rate of 80%, which was lower than in other studies [33,34] but similar to that reported by Conlisk et al. [30].

All implants in our series were CCK and not posterior stabilized. To our knowledge, there are no reports directly comparing stem type and fixation method in revision TKA.

Our study is subject to a series of limitations. First, clinical and radiological data were analyzed retrospectively. Second, the cohort was too small to determine the implant survival rate. Third, no control group was included. Finally, as ours is a major referral center, referral bias is possible. Although these factors might account for some of the differences in implant survival, our results were comparable to those published for knee revision using similar constrained systems.

The strengths of our study include the homogeneity of the approach used, namely, a single knee revision system, with a CCK liner in all cases. Furthermore, all the procedures were performed at a single institution by 2 senior surgeons, and postoperative data were collected using a uniform, controlled protocol through the joint registry; consequently, the likelihood of differential measurement bias is reduced. Nevertheless, a multicenter trial is necessary to ensure more robust results. Finally, our analysis combined both implant survival with long-term clinical outcome measures and patient-reported subjective measures to evaluate the performance of these implants.

To our knowledge, ours is the first study to provide encouraging results for range of movement, clinical scores, implant fixation stability, and freedom from complications with a follow-up of more than 5 years based on the same CCK arthroplasty system. Although no significant functional differences were recorded between the groups, further re-evaluations of these series could elucidate the potential long-term significance of the radiolucent lines, which are more frequent in the group with surface cementation.

## Conclusion

In conclusion, the modular, fixed-bearing LCCK TKA (Zimmer™) system has an acceptable long-term survival rate in both septic and aseptic revisions, although the frequency of complications is high. Metaphyseal cementation provides better radiologic outcome than surface cementation; however, the difference was not clinically significant. For patients with long-term follow-up and implant survival, values for objective clinical outcome measures and subjective patient-reported outcome measures were significantly better than the preoperative values.
